# Supplementary material for: Analysis of PPARγ Signaling Activity in Psoriasis
Source: Int J Mol Sci. 2021 Aug 10;22(16):8603. doi: 10.3390/ijms22168603 (PMC8395241; doi:10.3390/ijms22168603)
Supplement: Supplementary file 1 [file ijms-22-08603-s001.zip › Supplemental materials_Analysis of PPARg signaling activity in psoriasis/Pathway models/Models images and html files/Interleukin-17 and interleukin-22 signaling in psoriasis/2909.html]

CSF2


# Protein CSF2

|  |  |
| --- | --- |
| URN | urn:agi-llid:1437 |
| Total Entities | 0 |
| Connectivity | 6678 |
| Name | CSF2 |
| Description | colony stimulating factor 2 (granulocyte-macrophage) |
| Notes | The protein encoded by this gene is a cytokine that controls the production, differentiation, and function of granulocytes and macrophages. The active form of the protein is found extracellularly as a homodimer. This gene has been localized to a cluster of related genes at chromosome region 5q31, which is known to be associated with interstitial deletions in the 5q- syndrome and acute myelogenous leukemia. Other genes in the cluster include those encoding interleukins 4, 5, and 13. [provided by RefSeq, Jul 2008] |
| Primary Cell Localization | Extracellular |
| Class | Ligand |

---

|  |  |
| --- | --- |
| Pathway | Cortisol in Resolving Inflammation |
|  | Natural Killer Cell Activation through C-Type Lectin-like Receptors |
|  | Natural Killer Cell Activation through ITAM-Containing Receptors |
|  | Natural Killer Cell Activation through ITSM-Containing Receptors |
|  | Proplatelet Maturation |
|  | Lipids Enhance Apoptotic Cell Engulfment and Reduce Inflammation |
|  | Keep out Signal: LTF Inhibits Neutrophil Recruitment and Inflammation |
|  | Histamine Effects in Inflammation |
|  | Eosinophil Survival by Cytokine Signaling |
|  | Eosinophil Activation |
|  | Mast-Cells De Novo Synthesized Mediators via IgE Independent Signaling |
|  | Macrophage Related Iron Uptake and Release |
|  | Macrophage M1 Lineage |
|  | CD16/CD14 Proinflammatory Monocyte Activation |
|  | Neutrophil Recruitment and Priming |
|  | Myeloid Progenitor Cell -> GEMM - CFU Surface Expression Markers |
|  | Proteins Involved in Atherosclerosis |
|  | Proteins Involved in Myocarditis |
|  | Proteins Involved in Atopic Dermatitis |
|  | Proteins Involved in Melanoma |
|  | IL17 Signaling in Psoriasis |
|  | Proteins Involved in Psoriasis |
|  | Proteins Involved in Vitiligo |
|  | Proteins Involved in Diabetes Mellitus Type 1 |
|  | Hodgkin and Reed-Sternberg Cells in Hodgkin Lymphoma |
|  | Hodgkin Lymphoma |
|  | Eosinophil Survival in Asthma |
|  | Antigen-Presenting Cell Role in Asthma |
|  | Proteins Involved in Rheumatoid Arthritis |
|  | Proteins Involved in HIV Type 1 Infection |
|  | Proteins Involved in Alzheimer's Disease |
|  | Proteins Involved in Amyotrophic Lateral Sclerosis |
|  | Proteins Involved in Multiple Sclerosis |
|  | Subepithelial Fibroblasts in Middle Ear Cholesteatoma |
|  | Cholesteatoma |
|  | Glucocorticoid Resistance |
|  | Proteins Involved in Chronic Obstructive Pulmonary Disease |
|  | Alveolar Epithelial Cell Dysfunction in COPD |
|  | Proteins Involved in Glomerulonephritis |
|  | FCGR3A Expression Targets |
|  | CD8 -> ATF/CREB/CREBBP Expression Targets |
|  | CD80 -> AP-1 Expression Targets |
|  | CXCL12 Expression Targets |
|  | CSF2 -> NF-kB Expression Targets |
|  | CSF2 -> STAT Expression Targets |
|  | Clozapine Induced Granulocytopenia |
|  | EGF -> CTNN Expression Targets |
|  | EGF -> AP-1/ATF Expression Targets |
|  | EGF -> CREB/CREBBP/ELK/SRF/MYC Expression Targets |
|  | EGF -> MEF/MYOD/NFATC Expression Targets |
|  | EGF -> STAT Expression Targets |
|  | F2 -> AP-1/CREB/ELK/SRF/SP1 Expression Targets |
|  | F2 -> STAT1/NF-kB Expression Targets |
|  | FGF2 -> STAT Expression Targets |
|  | FGF2 -> AP-1/CREB/CREBBP/ELK/SRF/MYC Expression Targets |
|  | GH1/GHR -> NF-kB/ELK/SRF/MYC Expression Targets |
|  | GH1/GHR -> STAT Expression Targets |
|  | GH1/PRLR Expression Targets |
|  | TAM Receptor Signaling Activation in Cancer |
|  | Myeloid Derived Suppressor Cells in Cancer Immune Escape |
|  | Proteins with Altered Expression in Cancer Immune Escape |
|  | Fibronectin Expression Targets |
|  | EGFR -> Expression Targets in Skin |
|  | IL15 Expression Targets |
|  | IL3 Expression Targets |
|  | IL2 Expression Targets |
|  | PAF/Gq -> AP-1/ATF1/CREB/ERK/SRF Expression Targets |
|  | PGE1 Expression Targets |
|  | EGFR-> Expression Targets in Lymphoid System and Blood |
|  | NGF -> SMAD3/NF-kB Expression Targets |
|  | NGF -> AP-1/TP53/MYC Expression Targets |
|  | EDN1 Expression Targets |
|  | IFNA1/Gq Expression Targets |
|  | KITLG -> STAT Expression Targets |
|  | KITLG -> AP-1/CREB/CREBBP/MYC Expression Targets |
|  | PAF Expression Targets |
|  | TCR -> NF-kB Expression Targets |
|  | TCR -> NFAT Expression Targets |
|  | TCR -> AP-1 Expression Targets |
|  | TGFB2-TGFBR2 Expression Targets |
|  | TNF -> NF-kB Expression Targets |
|  | LTA Expression Targets |
|  | TNF -> AP-1 Expression Targets |
|  | TLR4 -> AP-1 Expression Targets |
|  | IL1B Expression Targets |
|  | IL1A Expression Targets |
|  | ROS in Triggering Vascular Inflammation |
|  | prarg neg,ukn expres targets, ps-positive |
|  | PPARG negative targets |
|  | pparg expr target\_regulators |
|  | Neighbors of disease exacerbation |
|  | Neighbors of tumor progression |
|  | Neighbors of cancer progression |
|  | Neighbors of inflammatory status |
|  | Neighbors of overall survival |
|  | Neighbors of disease severity |
|  | Neighbors of tumor size |
|  | Neighbors of lesion size |
|  | Neighbors of mortality |
|  | Neighbors of survival rate |
|  | Neighbors of infarct size |
|  | Neighbors of survival time |
|  | Neighbors of functional recovery |
|  | Neighbors of insulin sensitivity |
|  | Neighbors of bacterial load |
|  | Neighbors of cancer survival |
|  | Neighbors of graft survival |
|  | Neighbors of therapeutic efficacy |
|  | Neighbors of clinical stage |
|  | Neighbors of lethality |
|  | Neighbors of tumor regression |
|  | Neighbors of recurrence-free survival |
|  | Neighbors of disease-free survival |
|  | Neighbors of tumor response |
|  | Neighbors of macrophage count |
|  | Neighbors of long-term survival |
|  | Neighbors of treatment outcome |
|  | Neighbors of body weight |
|  | Neighbors of microvessel density |
|  | Neighbors of tumor promotion |
|  | Neighbors of neutrophil count |
|  | Neighbors of morbidity |
|  | Neighbors of fibrosis progression |
|  | Neighbors of mortality rate |
|  | Neighbors of fat mass |
|  | Neighbors of exhaustion |
|  | Neighbors of adverse outcome |
|  | Neighbors of lesion area |
|  | Neighbors of cell density |
|  | Neighbors of progression-free survival |
|  | Neighbors of apoptosis rate |
|  | Neighbors of viral load |
|  | Neighbors of leukocyte count |
|  | Neighbors of vascular density |
|  | Neighbors of muscle mass |
|  | Neighbors of ejection fraction |
|  | Neighbors of median survival |
|  | Neighbors of osteoclast number |
|  | Neighbors of cancer recurrence |
|  | Neighbors of T-cell count |
|  | Neighbors of antimetastatic activity |
|  | Neighbors of energy expenditure |
|  | Neighbors of glucose level |
|  | Neighbors of left ventricular ejection fraction |
|  | Neighbors of proliferation index |
|  | Neighbors of cell permeability |
|  | Neighbors of pregnancy outcome |
|  | Neighbors of functional status |
|  | Neighbors of neurological outcome |
|  | Neighbors of fertility |
|  | Neighbors of recurrence rate |
|  | Neighbors of lymphocyte count |
|  | Neighbors of monocyte count |
|  | Neighbors of insulin level |
|  | Neighbors of disease exacerbation |
|  | Neighbors of tumor progression |
|  | Neighbors of cancer progression |
|  | Neighbors of inflammatory status |
|  | Neighbors of overall survival |
|  | Neighbors of disease severity |
|  | Neighbors of tumor size |
|  | Neighbors of lesion size |
|  | Neighbors of mortality |
|  | Neighbors of survival rate |
|  | Neighbors of infarct size |
|  | Neighbors of survival time |
|  | Neighbors of functional recovery |
|  | Neighbors of insulin sensitivity |
|  | Neighbors of bacterial load |
|  | Neighbors of cancer survival |
|  | Neighbors of graft survival |
|  | Neighbors of therapeutic efficacy |
|  | Neighbors of clinical stage |
|  | Neighbors of lethality |
|  | Neighbors of tumor regression |
|  | Neighbors of recurrence-free survival |
|  | Neighbors of disease-free survival |
|  | Neighbors of tumor response |
|  | Neighbors of macrophage count |
|  | Neighbors of long-term survival |
|  | Neighbors of treatment outcome |
|  | Neighbors of body weight |
|  | Neighbors of microvessel density |
|  | Neighbors of tumor promotion |
|  | Neighbors of neutrophil count |
|  | Neighbors of morbidity |
|  | Neighbors of fibrosis progression |
|  | Neighbors of mortality rate |
|  | Neighbors of fat mass |
|  | Neighbors of exhaustion |
|  | Neighbors of adverse outcome |
|  | Neighbors of lesion area |
|  | Neighbors of cell density |
|  | Neighbors of progression-free survival |
|  | Neighbors of apoptosis rate |
|  | Neighbors of viral load |
|  | Neighbors of leukocyte count |
|  | Neighbors of vascular density |
|  | Neighbors of muscle mass |
|  | Neighbors of ejection fraction |
|  | Neighbors of median survival |
|  | Neighbors of osteoclast number |
|  | Neighbors of cancer recurrence |
|  | Neighbors of T-cell count |
|  | Neighbors of antimetastatic activity |
|  | Neighbors of energy expenditure |
|  | Neighbors of glucose level |
|  | Neighbors of left ventricular ejection fraction |
|  | Neighbors of proliferation index |
|  | Neighbors of cell permeability |
|  | Neighbors of pregnancy outcome |
|  | Neighbors of functional status |
|  | Neighbors of neurological outcome |
|  | Neighbors of fertility |
|  | Neighbors of recurrence rate |
|  | Neighbors of lymphocyte count |
|  | Neighbors of monocyte count |
|  | Neighbors of insulin level |
|  | Neighbors of cell development |
|  | Neighbors of immune response |
|  | Neighbors of cell population |
|  | Neighbors of T-cell development |
|  | Neighbors of inflammatory response |
|  | Neighbors of wound healing |
|  | Neighbors of angiogenesis |
|  | Neighbors of innate immune response |
|  | Neighbors of cell function |
|  | Neighbors of macrophage activation |
|  | Neighbors of cell formation |
|  | Neighbors of adaptive immune response |
|  | Neighbors of osteoclast development |
|  | Neighbors of epithelial to mesenchymal transition |
|  | Neighbors of immunity |
|  | Neighbors of cell proliferative response |
|  | Neighbors of chemotaxis |
|  | Neighbors of dendritic cell differentiation |
|  | Neighbors of cell survival |
|  | Neighbors of osteoclast differentiation |
|  | Neighbors of cell phenotype |
|  | Neighbors of T-cell proliferation |
|  | Neighbors of tissue remodeling |
|  | Neighbors of cell adhesion |
|  | Neighbors of T-cell activation |
|  | Neighbors of endothelial cell proliferation |
|  | Neighbors of fibrogenesis |
|  | Neighbors of tumor growth |
|  | Neighbors of dendritic cell development |
|  | Neighbors of tissue repair |
|  | Neighbors of cell count |
|  | Neighbors of cell infiltration |
|  | Neighbors of cell interaction |
|  | Neighbors of SMC proliferation |
|  | Neighbors of lumen formation |
|  | Neighbors of endothelial cell migration |
|  | Neighbors of adipogenesis |
|  | Neighbors of lipid storage |
|  | Neighbors of cell damage |
|  | Neighbors of pregnancy |
|  | Neighbors of monocyte differentiation |
|  | Neighbors of aging |
|  | Neighbors of cell motility |
|  | Neighbors of bone resorption |
|  | Neighbors of monocyte migration |
|  | Neighbors of fibroblast proliferation |
|  | Neighbors of macrophage migration |
|  | Neighbors of microglial activation |
|  | Neighbors of neutrophil recruitment |
|  | Neighbors of regeneration |
|  | Neighbors of cell migration |
|  | Neighbors of cancer growth |
|  | Neighbors of stem cell proliferation |
|  | Neighbors of hepatic regeneration |
|  | Neighbors of transendothelial migration |
|  | Neighbors of phagocytosis |
|  | Neighbors of immune system activation |
|  | Neighbors of leukocyte migration |
|  | Neighbors of leukocyte recruitment |
|  | Neighbors of osteoclast formation |
|  | Neighbors of monocyte recruitment |
|  | Neighbors of cell invasion |
|  | Neighbors of heart function |
|  | Neighbors of hemopoiesis |
|  | Neighbors of bone remodeling |
|  | Neighbors of ossification |
|  | Neighbors of T-cell function |
|  | Neighbors of immune system function |
|  | Neighbors of lung development |
|  | Neighbors of autophagy |
|  | Neighbors of monocyte adhesion |
|  | Neighbors of transcription activation |
|  | Neighbors of senescence |
|  | Neighbors of T-cell response |
|  | Neighbors of tumor immunity |
|  | Neighbors of hepatocyte proliferation |
|  | Neighbors of cell transdifferentiation |
|  | Neighbors of neuroprotection |
|  | Neighbors of stem cell differentiation |
|  | Neighbors of endothelial cell function |
|  | Neighbors of neuronal death |
|  | Neighbors of cell growth |
|  | Neighbors of ROS generation |
|  | Neighbors of epithelial cell proliferation |
|  | Neighbors of first trimester pregnancy |
|  | Neighbors of cancer cell growth |
|  | Neighbors of sensitization |
|  | Neighbors of hemato-encephalic barrier |
|  | Neighbors of macrophage function |
|  | Neighbors of cellular immune response |
|  | Neighbors of cellular senescence |
|  | Neighbors of colony formation |
|  | Neighbors of macrophage differentiation |
|  | Neighbors of cell differentiation |
|  | Neighbors of keratinocyte proliferation |
|  | Neighbors of stem cell migration |
|  | Neighbors of life span |
|  | Neighbors of cell homeostasis |
|  | Neighbors of psoriasis |
|  | Neighbors of inflammatory disease |
|  | Neighbors of rheumatoid arthritis |
|  | Neighbors of atherosclerosis |
|  | Neighbors of fibrosis |
|  | Neighbors of colitis |
|  | Neighbors of arthritis |
|  | Neighbors of inflammatory bowel disease |
|  | Neighbors of injury |
|  | Neighbors of atherogenesis |
|  | Neighbors of vascular remodeling |
|  | Neighbors of metastasis |
|  | Neighbors of carcinogenesis |
|  | Neighbors of multiple sclerosis |
|  | Neighbors of chronic inflammation |
|  | Neighbors of inflammation |
|  | Neighbors of tumor microenvironment |
|  | Neighbors of pathological angiogenesis |
|  | Neighbors of obesity |
|  | Neighbors of vascular disease |
|  | Neighbors of diabetes mellitus |
|  | Neighbors of asthma |
|  | Neighbors of pneumonia |
|  | Neighbors of hyperplasia |
|  | Neighbors of osteoarthritis |
|  | Neighbors of liver fibrosis |
|  | Neighbors of chronic obstructive pulmonary disease |
|  | Neighbors of myocardial infarction |
|  | Neighbors of leukocyte infiltration |
|  | Neighbors of reperfusion injury |
|  | Neighbors of airway inflammation |
|  | Neighbors of insulin resistance |
|  | Neighbors of infection |
|  | Neighbors of vascular injury |
|  | Neighbors of renal injury |
|  | Neighbors of macrophage infiltration |
|  | Neighbors of liver injury |
|  | Neighbors of neuroinflammation |
|  | Neighbors of sepsis |
|  | Neighbors of death |
|  | Neighbors of type 1 diabetes |
|  | Neighbors of encephalomyelitis |
|  | Neighbors of autoimmune disease |
|  | Neighbors of ischemic stroke |
|  | Neighbors of dermatitis |
|  | Neighbors of neutrophil infiltration |
|  | Neighbors of heart disease |
|  | Neighbors of lung injury |
|  | Neighbors of immunopathology |
|  | Neighbors of systemic sclerosis |
|  | Neighbors of hepatocellular carcinoma |
|  | Neighbors of autoimmunity |
|  | Neighbors of acute lung injury |
|  | Neighbors of gastric cancer |
|  | Neighbors of experimental autoimmune encephalomyelitis |
|  | Neighbors of colorectal cancer |
|  | Neighbors of cardiac remodeling |
|  | Neighbors of breast cancer |
|  | Neighbors of ischemia |
|  | Neighbors of systemic lupus erythematosus |
|  | Neighbors of melanoma |
|  | Neighbors of collagen-induced arthritis |
|  | Neighbors of cartilage degeneration |
|  | Neighbors of neoplasm invasion |
|  | Neighbors of kidney disease |
|  | Neighbors of heart failure |
|  | Neighbors of lung disease |
|  | Neighbors of pulmonary fibrosis |
|  | Neighbors of cardiovascular disease |
|  | Neighbors of stroke |
|  | Neighbors of cancer |
|  | Neighbors of cerebral ischemia |
|  | Neighbors of type 2 diabetes |
|  | Neighbors of neoplasm |
|  | Neighbors of neointima |
|  | Neighbors of vasculitis |
|  | Neighbors of respiratory hypersensitivity |
|  | Neighbors of synovitis |
|  | Neighbors of coronary artery disease |
|  | Neighbors of rupture |
|  | Neighbors of endothelial cell dysfunction |
|  | Neighbors of liver disease |
|  | Neighbors of prostate cancer |
|  | Neighbors of hypertrophy |
|  | Neighbors of osteolysis |
|  | Neighbors of ovarian cancer |
|  | Neighbors of pancreatic cancer |
|  | Neighbors of allograft rejection |
|  | Neighbors of epithelial cell |
|  | Neighbors of keratinocyte |
|  | Neighbors of vascular smooth muscle cell |
|  | Neighbors of umbilical vein endothelial cell |
|  | Neighbors of monocyte |
|  | Neighbors of endometrial stromal cell |
|  | Neighbors of alveolar macrophage |
|  | Neighbors of synoviocyte |
|  | Neighbors of macrophage |
|  | Neighbors of adipocyte |
|  | Neighbors of activated monocyte |
|  | Neighbors of fibroblast |
|  | Neighbors of airway epithelial cell |
|  | Neighbors of PBMC |
|  | Neighbors of stromal cell |
|  | Neighbors of airway smooth muscle cell |
|  | Neighbors of osteoblast |
|  | Neighbors of pneumocyte |
|  | Neighbors of endometrium cell |
|  | Neighbors of microglia |
|  | Neighbors of trophoblast |
|  | Neighbors of intestine epithelium cell |
|  | Neighbors of mononuclear cell |
|  | Neighbors of hepatic stellate cell |
|  | Neighbors of hepatocyte |
|  | Neighbors of decidual cell |
|  | Neighbors of endothelial cell |
|  | Neighbors of dendritic cell |
|  | Neighbors of Kuppfer cell |
|  | Neighbors of proadipocyte |
|  | Neighbors of kidney tubule cell |
|  | Neighbors of inflammatory cell |
|  | Neighbors of bone marrow macrophage |
|  | Neighbors of peritoneal macrophage |
|  | Neighbors of osteoclast |
|  | Neighbors of neutrophil |
|  | Neighbors of smooth muscle myocyte |
|  | Neighbors of astrocyte |
|  | Neighbors of bone marrow derived dendritic cell |
|  | Neighbors of lung fibroblast |
|  | Neighbors of activated T-cell |
|  | Neighbors of mesothelial cell |
|  | Neighbors of mononuclear phagocyte |
|  | Neighbors of capillary endothelial cell |
|  | Neighbors of immunocompetent cell |
|  | Neighbors of granulocyte |
|  | Neighbors of cancer-associated fibroblast |
|  | Neighbors of myeloid cell |
|  | Neighbors of mesenchymal stroma cell |
|  | Neighbors of corneal epithelial cell |
|  | Neighbors of cytotrophoblast |
|  | Neighbors of stem cell |
|  | Neighbors of tumor infiltrating macrophage |
|  | Neighbors of B-cell |
|  | Neighbors of leukocyte |
|  | Neighbors of chondrocyte |
|  | Neighbors of insulin-secreting cell |
|  | Neighbors of mesenchymal cell |
|  | Neighbors of T-cell |
|  | Neighbors of M1 macrophage |
|  | Neighbors of myofibroblast |
|  | Neighbors of pancreatic stellate cell |
|  | Neighbors of mast cell |
|  | Neighbors of bone marrow stromal cell |
|  | Neighbors of spleen cell |
|  | Neighbors of brain capillary endothelial cell |
|  | Neighbors of neuroglia |
|  | Neighbors of thyroid epithelial cell |
|  | Neighbors of hematopoietic cell |
|  | Neighbors of podocyte |
|  | Neighbors of conventional dendritic cell |
|  | Neighbors of antigen-presenting cell |
|  | Neighbors of epidermis cell |
|  | Neighbors of bone marrow derived mast cell |
|  | Neighbors of phagocyte |
|  | Neighbors of kidney cell |
|  | Neighbors of lymphocyte |
|  | Neighbors of Langerhans cell |
|  | Neighbors of decidual natural killer cell |
|  | Neighbors of thymic epithelial cell |
|  | Neighbors of M2 macrophage |
|  | Neighbors of lymphoid cell |
|  | Neighbors of blood cell |
|  | Neighbors of skin cell |
|  | Neighbors of peripheral lymphocyte |
|  | Neighbors of activated B-cell |
|  | Neighbors of lymph node cell |
|  | Neighbors of lung |
|  | Neighbors of liver |
|  | Neighbors of kidney |
|  | Neighbors of skin |
|  | Neighbors of intestine |
|  | Neighbors of lymph node |
|  | Neighbors of colon |
|  | Neighbors of airway |
|  | Neighbors of blood vessel |
|  | Neighbors of heart |
|  | Neighbors of aorta |
|  | Neighbors of microvessel |
|  | Neighbors of bone |
|  | Neighbors of placenta |
|  | Neighbors of synovial membrane |
|  | Neighbors of uterus |
|  | Neighbors of retina |
|  | Neighbors of ovary |
|  | Neighbors of breast |
|  | Neighbors of artery |
|  | Neighbors of pancreas |
|  | Neighbors of prostate |
|  | Neighbors of central nervous system |
|  | Neighbors of lymphatic system |
|  | Neighbors of colorectal region |
|  | Neighbors of decidua |
|  | Neighbors of spleen |
|  | Neighbors of peritoneum |
|  | Neighbors of allantois |
|  | Neighbors of alveolus |
|  | Neighbors of left ventricle |
|  | Neighbors of uterine cervix |
|  | Neighbors of vein |
|  | Neighbors of thymus gland |
|  | Neighbors of myelin sheath |
|  | Neighbors of draining lymph node |
|  | Neighbors of nervous system |
|  | Neighbors of trachea |
|  | Neighbors of ileum |
|  | Neighbors of small intestine |
|  | Neighbors of aggregated lymphoid follicle of small intestine |
|  | Neighbors of NF-kB family |
|  | Neighbors of oxidized LDL |
|  | Neighbors of JNK |
|  | Neighbors of IL1 family |
|  | Neighbors of PPAR |
|  | Neighbors of Jun/Fos |
|  | Neighbors of NFKBI |
|  | Neighbors of PKC |
|  | Neighbors of STAT family |
|  | Neighbors of mitogen-activated protein kinase |
|  | Neighbors of IFNAR ligand |
|  | Neighbors of transforming growth factor |
|  | Neighbors of TLR |
|  | Neighbors of cytokine |
|  | Neighbors of interferon |
|  | Neighbors of interleukin |
|  | Neighbors of JAK |
|  | Neighbors of MEK1/2 |
|  | Neighbors of PDGF |
|  | Neighbors of ERK1/2 |
|  | Neighbors of growth factor |
|  | Neighbors of ovalbumin |
|  | Neighbors of LDL |
|  | Neighbors of NF-AT family |
|  | Neighbors of beta adrenoceptor |
|  | Neighbors of IKBK |
|  | Neighbors of protein tyrosine kinase |
|  | Neighbors of SMAD subfamily |
|  | Neighbors of AMPK |
|  | Neighbors of p38 |
|  | Neighbors of inflammatory cytokine |
|  | Neighbors of IL23 |
|  | Neighbors of T-cell receptor |
|  | Neighbors of histone deacetylase |
|  | Neighbors of IL1R |
|  | Neighbors of IL12 |
|  | Neighbors of prostaglandin-endoperoxide synthase |
|  | Neighbors of heat shock protein 90 |
|  | Neighbors of lectin |
|  | Neighbors of mitogen-activated protein kinase kinase |
|  | Neighbors of proteasome endopeptidase complex |
|  | Neighbors of histone H3 |
|  | Neighbors of STAT5 |
|  | Neighbors of retinoid-X receptor subfamily |
|  | Neighbors of PP2B |
|  | Neighbors of CD3 complex |
|  | Neighbors of TLR1/2 heterodimer |
|  | Neighbors of PDGF receptor |
|  | Neighbors of pancreatic elastase |
|  | Neighbors of histone acetyltransferase |
|  | 2\_Interleukin-17 and interleukin-22 signaling in psoriasis |
|  | 1\_Cigarette smoke causes dysfunction and production of inflammatory mediators in alveolar epithelial cells |
|  | 1\_Th2 cell response in asthma |
|  | 2\_2\_Eosinophilia and neutrophilia in asthma: eosinophil apoptosis |
|  | 6\_p12I Viral protein promotes T-cell activation in HTLV-1 infection |
|  | drug-target moltransport |
|  | drug-target molsynthesis |
|  | Interleukin-17 and interleukin-22 signaling in psoriasis |

---

|  |  |
| --- | --- |
| Group | Genes with Mutations Associated with Atopic Dermatitis |

---

|  |  |
| --- | --- |
| MedScan ID | 1437 |

---

|  |  |
| --- | --- |
| LocusLink ID | 1437 |
|  | 12981 |
|  | 116630 |

---

|  |  |
| --- | --- |
| Alias | granulocyte-macrophage colony stimulating factor |
|  | CSF2 |
|  | GM-CSF |
|  | granulocyte-macrophage colony stimulation factor |
|  | OTTMUSP00000005830 |
|  | colony stimulating factor 2 |
|  | colony stimulating factor 2 (granulocyte-macrophage) |
|  | MGC138897 |
|  | granulocyte macrophage colony-stimulating factor receptor ligands |
|  | RP23-309E16.1 |
|  | Csfgm |
|  | colony stimulation factor 2 (granulocyte-macrophage) |
|  | GMCSF |
|  | CSF2\_HUMAN |
|  | put. GM-CSF |
|  | colony stimulation factor 2 |
|  | MGI-IGM |
|  | granulocyte-macrophage colony stimulating factor 2 |
|  | GM-CSF receptor ligand |
|  | MGC151255 |
|  | MGC151257 |
|  | MGC131935 |
|  | granulocyte macrophage colony-stimulating factor receptor ligand |
|  | CSF |
|  | granulocyte macrophage-colony stimulating factor |
|  | molgramostim |
|  | molgramostin |
|  | sargramostim |
|  | granulocyte-macrophage colony-stimulating factor |
|  | colony-stimulating factor |
|  | rhu gm-csf |
|  | leukine |

---

|  |  |
| --- | --- |
| GO ID | 0005125 |
|  | 0005129 |
|  | 0008083 |
|  | 0000165 |
|  | 0097011 |
|  | 0071222 |
|  | 0019221 |
|  | 0097028 |
|  | 0001892 |
|  | 0042045 |
|  | 0001821 |
|  | 0006955 |
|  | 0042116 |
|  | 0030225 |
|  | 0030224 |
|  | 0043011 |
|  | 0045918 |
|  | 2001240 |
|  | 0045892 |
|  | 0030223 |
|  | 0008284 |
|  | 0010628 |
|  | 0032747 |
|  | 0010744 |
|  | 0071803 |
|  | 0042531 |
|  | 0045187 |
|  | 0045637 |
|  | 0034405 |
|  | 0034021 |
|  | 0005576 |
|  | 0005615 |
|  | 0043231 |
|  | 0042127 |
|  | 0010468 |
|  | 0070371 |
|  | 0031929 |
|  | 0050673 |
|  | 0072651 |
|  | 0014065 |
|  | 0045651 |
|  | 1904075 |
|  | 0043491 |
|  | 0010469 |
|  | 0005088 |
|  | 0004713 |
|  | 0018108 |
|  | 0045740 |
|  | 0043547 |
|  | 0042523 |
|  | 0005622 |
|  | 0043066 |
|  | 0045885 |

---

|  |  |
| --- | --- |
| KEGG ID | rno:116630 |
|  | hsa:1437 |
|  | mmu:12981 |

---

|  |  |
| --- | --- |
| Organism | Homo sapiens {Organism urn:agi-taxid:9606} |
|  | Mus musculus {Organism urn:agi-taxid:10090} |
|  | Rattus norvegicus {Organism urn:agi-taxid:10116} |
|  | Homo sapiens |
|  | Mus musculus |
|  | Rattus norvegicus |

---

|  |  |
| --- | --- |
| Mouse chromosome position | 11 32.13 cM |
|  | 11 29.5 cM |

---

|  |  |
| --- | --- |
| OMIM ID | 138960 |

---

|  |  |
| --- | --- |
| Rat chromosome position | 10q22 |

---

|  |  |
| --- | --- |
| Hugo ID | 2434 |
|  | HGNC:2434 |

---

|  |  |
| --- | --- |
| Human chromosome position | 5q31.1 |

---

|  |  |
| --- | --- |
| Swiss-Prot Accession | P04141 |
|  | P04141.1 |
|  | P01587 |
|  | Q5SX78 |
|  | P01587.1 |
|  | P48750 |
|  | P48750.2 |
|  | Q14CE8 |
|  | Q2VPI8 |
|  | Q8NFI6 |
|  | Q647J8 |
|  | Q14AD9 |

---

|  |  |
| --- | --- |
| PIR ID | I46269 |
|  | C24636 |
|  | I48368 |

---

|  |  |
| --- | --- |
| GenBank ID | NC\_000005 |
|  | NM\_000758 |
|  | NP\_000749 |
|  | NG\_033024 |
|  | AC004511 |
|  | AAC08707 |
|  | AC034228 |
|  | AF373868 |
|  | AAK51563 |
|  | CH471062 |
|  | EAW62353 |
|  | CS401638 |
|  | CAL40350 |
|  | HC025839 |
|  | CBH19850 |
|  | HI574012 |
|  | CBX47495 |
|  | M13207 |
|  | AAA98768 |
|  | X03021 |
|  | CAA26822 |
|  | AF510855 |
|  | AAM44054 |
|  | AY720441 |
|  | AAU21240 |
|  | BC108724 |
|  | AAI08725 |
|  | BC113924 |
|  | AAI13925 |
|  | BC113999 |
|  | AAI14000 |
|  | KP271009 |
|  | AJC19278 |
|  | M10663 |
|  | AAA52121 |
|  | M11220 |
|  | AAA52578 |
|  | M11734 |
|  | AAA52122 |
|  | P04141 |
|  | NC\_000077 |
|  | NM\_009969 |
|  | NP\_034099 |
|  | XM\_006532127 |
|  | XP\_006532190 |
|  | AL596103 |
|  | CH466575 |
|  | EDL33540 |
|  | X03020 |
|  | CAA26821 |
|  | AK053196 |
|  | BAE20692 |
|  | BC116878 |
|  | AAI16879 |
|  | BC116880 |
|  | AAI16881 |
|  | EU366957 |
|  | ABY66391 |
|  | M11848 |
|  | AAA37483 |
|  | X02333 |
|  | CAA26193 |
|  | X03019 |
|  | CAA26820 |
|  | X03221 |
|  | X05906 |
|  | CAA29336 |
|  | P01587 |
|  | NC\_005109 |
|  | NM\_053852 |
|  | NP\_446304 |
|  | AC\_000078 |
|  | AABR07029603 |
|  | AAHX01063930 |
|  | CH473948 |
|  | EDM04426 |
|  | U00620 |
|  | AAA18281 |
|  | P48750 |
|  | NC\_018916 |
|  | AMYH02012209 |
|  | AC\_000033 |
|  | AAHY01093388 |
|  | AC\_000137 |
|  | ABBA01068814 |
|  | CAI25072 |
|  | XM\_340799 |
|  | XP\_340800 |
|  | XM\_001074265 |
|  | XP\_001074265 |
|  | NT\_034772 |
|  | NW\_001838952 |
|  | NW\_922784 |
|  | AC\_000048 |
|  | Q647J8 |
|  | NT\_096135 |
|  | NW\_001030461 |
|  | Q14AD9 |
|  | Q5SX78 |
|  | NW\_001084656 |
|  | NW\_047334 |

---

|  |  |
| --- | --- |
| Swiss-Prot ID | CSF2\_RAT |
|  | CSF2\_HUMAN |
|  | CSF2\_MOUSE |
|  | Q5SX78\_MOUSE |

---

|  |  |
| --- | --- |
| Cell Localization | Secreted |
|  | Extracellular |

---

|  |  |
| --- | --- |
| Ensembl ID | ENSG00000164400 |
|  | ENSP00000296871.2 |
|  | ENST00000296871.4 |
|  | ENSMUSG00000018916 |
|  | ENSMUSP00000019060.5 |
|  | ENSMUST00000019060.5 |
|  | ENSRNOG00000026805 |
|  | ENSRNOP00000034235.3 |
|  | ENSRNOT00000032333.3 |
|  | ENSP00000296871 |
|  | ENST00000296871 |
|  | ENSMUSP00000019060 |
|  | ENSMUST00000019060 |
|  | ENSRNOP00000034235 |
|  | ENSRNOT00000032333 |

---

|  |  |
| --- | --- |
| MGI ID | MGI:1339752 |
|  | 1339752 |

---

|  |  |
| --- | --- |
| RGD ID | 621065 |

---

|  |  |
| --- | --- |
| Unigene ID | Hs.1349 |
|  | Mm.4922 |
|  | Rn.44285 |

---

|  |  |
| --- | --- |
| Homologene ID | 600 |

---

|  |  |
| --- | --- |
| Shape | Rhomb |

---

|  |  |
| --- | --- |
| IPI ID | IPI00323907 |
|  | IPI00022270 |
|  | IPI00372574 |

---
